# Influence of acute progressive hypoxia on cardiovascular variability in conscious spontaneously hypertensive rats

**DOI:** 10.1016/j.autneu.2008.05.008

**Published:** 2008-08-18

**Authors:** Mitsutaka Sugimura, Yohsuke Hirose, Hiroshi Hanamoto, Kenji Okada, Aiji Boku, Yoshinari Morimoto, Kunitaka Taki, Hitoshi Niwa

**Affiliations:** Department of Dental Anesthesiology, Graduate School of Dentistry, Osaka University, 1–8 Yamadaoka, Suita, Osaka 565-0871, Japan

**Keywords:** Acute progressive hypoxia, Autonomic nervous activity, Variability, Striatal dopamine, Spontaneous hypertensive rats, Wavelet

## Abstract

The purpose of this study is to examine the influence of acute progressive hypoxia on cardiovascular variability and striatal dopamine (DA) levels in conscious, spontaneously hypertensive rats (SHR) and Wistar Kyoto rats (WKY). After preparation for measurement, the inspired oxygen concentration of rats was decreased to 10% within 5 min (descent stage), maintained at 10% for 10 min (fixed stage), and then elevated back to 20% over 5 min (recovery stage). The systolic blood pressure (SBP) and heart rate (HR) variability at each stage was calculated to evaluate the autonomic nervous system response using the wavelet method. Striatal DA during each stage was measured using in vivo microdialysis. We found that SHR showed a more profound hemodynamic response to progressive hypoxia as compared to WKY. Cardiac parasympathetic activity in SHR was significantly inhibited by acute progressive hypoxia during all stages, as shown by the decrease in the high frequency band of HR variability (HR-HF), along with transient increase in sympathetic activity during the early hypoxic phase. This decrease in the HR-HF continued even when SBP was elevated. Striatal DA levels showed the transient similar elevation in both groups. These findings suggest that acute progressive hypoxic stress in SHR inhibits cardiac parasympathetic activity through reduction of baroreceptor reflex sensitivity, with potentially severe deleterious effects on circulation, in particular on HR and circulatory control. Furthermore, it is thought that the influence of acute progressive hypoxia on striatal DA levels is similar in SHR and WKY.

## 1 Introduction

Hypoxia is one of the most potent stressors to the body. Diminution of oxygen tension in inspired gas not only stimulates ventilation through activation of the respiratory center and chemoreceptors, but also affects the autonomic nervous system (ANS).

Several animal studies conducted to assess the cardiovascular autonomic responses to hypoxia have shown variable results, probably due to different experimental settings such as the type and severity of hypoxic exposure and the method and depth of anesthesia. (Murasato et al., 1998; Hirakawa et al., 1997; Fukuda et al., 1989; Marshall and Metcalfe, 1988; Kawaguchi et al., 2005). Marshall and Metcalfe (1988) reported that a 3-min period of graded systemic hypoxia obtained by decreasing the fraction of inspired oxygen (F_I_O_2_) from 0.21 to 0.03 caused a fall in mean arterial pressure with a transient increase in heart rate (HR) in spontaneously breathing anesthetized rats. However, we believe that it is difficult to determine the isolated influence of acute hypoxia on the ANS under general anesthesia. In contrast, Kawaguchi et al. (2005) showed that chronic hypoxia with an F_I_O_2_ of 0.1 for 3 weeks caused attenuation of sympathetic nervous system activity and enhancement of parasympathetic nervous system activity in conscious rats. Their study did not capture the effects of moment-to-moment changes in FiO_2_ as occurs with acute progressive hypoxia in the conscious state. Although most studies have investigated the effects of acute hypoxia under anesthesia or chronic hypoxia in the conscious state as stated above, the mechanism by which the ANS responds to acute progressive hypoxia in conscious rats, particularly in hypertensive animals, is not yet understood.

Moreover, although it has been shown that central nervous system (CNS) activity is sensitive and vulnerable to hypoxic stress (Smith et al., 1984; Banasiak et al., 2000; Tuor et al., 2001), the time course of changes in CNS activity during acute progressive hypoxia is poorly understood. Furthermore, it has been reported that brain dopamine (DA) level, especially in the striatum and hippocampus, increases under oxygen-deprived conditions and the increase of DA levels induces harmful effects, including abetment of irreversible neural death (Nakajima et al., 1996; Busto et al., 1985; Globus et al., 1988; Globus et al., 1987; Masuda and Ito, 1993; Smith et al., 1984; Hedner and Lundborg, 1979). It has also been shown that increase of DA could lead to motor disturbances, behavioral abnormalities and learning disabilities over the long run (Lun et al., 1986; Erinoff et al., 1979; Hagberg et al., 1981). Based on these observations, it is considered that striatal DA is an indicator of hypoxic stress.

The purpose of this study was to examine the influence of acute progressive hypoxia on cardiovascular variability and striatal dopamine (DA) levels in Wistar Kyoto rats (WKY) and spontaneously hypertensive rats (SHR). The present study was designed to minimize surgical stress and was performed using freely moving conscious rats to determine the isolated influence of acute hypoxia on ANS activity and DA levels. Arterial BP and HR variability were calculated to evaluate the autonomic response, although the quantitative estimation method we used is somewhat controversial.

We used power spectral analysis with the wavelet (WT) method to estimate cardiovascular variability in this study because of its high time resolution (Akselrod et al., 1981; Kochs et al., 2001; Suzuki et al., 2003; Pichot et al., 1999; Ivanov et al., 1996). Though fast Fourier transform (FFT) has been used to analyze variability universally, it does not offer an easy assessment of transient autonomic alterations because of limitations inherent to stationary signals. WT is a method of resolving these limitations in conventional frequency-domain analysis such as FFT because this method does not require stability. Therefore, we believe that analyzing HR-HF and SBP-LF by the WT method can be used to assess rapid and complex fluctuations in the ANS during acute progressive hypoxia (Kawaguchi et al., 2005). Striatal DA, that was used just as an index of the hypoxic stress in the CNS, was measured using in vivo microdialysis and subsequent high performance liquid chromatography with electrochemical detection (HPLC-ECD) for 5-min on-line monitoring, which had been previously impossible (Nakajima et al., 1996; Globus et al., 1988).

## 2 Materials and methods

### 2.1 Animals and housing

The study protocol for animal experiments was approved by the Institute of Experimental Animal Sciences, Osaka University Graduate School of Dentistry, and carried out in accordance with the National Institutes of Health Guide for the Care and Use of Laboratory Animals (NIH Publications No. 80-23), revised in 1996. All efforts were made to minimize the number of animals used and their suffering.

Twelve-week-old male SHR, as the study group, and WKY serving as control (*n* = 12 in each group, each weighing 250–300 g, Japan SLC, Shizuoka, Japan) were housed in individual cages, with food and water available *ad libitum,* under a 12-h light-dark cycle (lights on at 7:00 a.m.) at a constant temperature of 23 +/− 1 °C.

### 2.2 Surgical preparation

Rats were anesthetized with sodium pentobarbital (50 mg/kg, intraperitoneally, Schering–Plough Animal Health Co., Tokyo, Japan), and a catheter was inserted into the femoral artery for continuous measurement of blood pressure (BP). The distal end of the catheter was then tunneled subcutaneously and pulled out from the dorsal aspect of the neck for data sampling under free-moving, wakeful conditions**.** Heparinization for three days, from the time of catheter insertion to the day of the experiment, was used to maintain catheter patency (Nagai et al., 2003). After arterial catheterization, the animals were placed in a stereotaxic apparatus (SR-6, NARISHIGE™, Tokyo, Japan), and a guide cannula (AG-8 type™, Eicom, Kyoto, Japan) for in vivo microdialysis and measurement of striatal DA levels was implanted into the right dorsal striatum of the brain with the following coordinates relative to the bregma: A + 0.2 mm; R + 2.8 mm; V − 3.4 mm, according to the atlas of Paxinos and Watson (1986). Throughout the surgical and study periods, and the interval in between, there were no significant gains and losses of weight in the rats.

### 2.3 Experimental procedures

All experiments were commenced at 9 a.m. to minimize circadian fluctuations. After recovering from instrumentation for 72 h, the stylet from the guide cannula was removed and replaced by a dialysis probe (A-I-8-02 type™, Eicom, Kyoto, Japan), the probe being secured to the guide cannula with a screw, and perfused with modified Ringer's solution (NaCl 147 mM, CaCl_2_ 1.2 mM, MgCl_2_ 1.1 mM; pH 7.4) at a rate of 2.0 ml/min. The outflow was connected by Teflon tubing to a high performance liquid chromatography with electrochemical detection system (HPLC-ECD) (HTEC-500™, Eicom, Kyoto, Japan) for 5-min on-line monitoring of DA levels from the dorsal striatum. The arterial catheter was connected to BP measuring equipment (Pressure monitoring kit, LIFE KIT™, NIHON KOHDEN, Tokyo, Japan). Each rat was then placed in a transparent, brownish-red, airtight, acrylic chamber (30 × 30 × 50 cm) that had two holes, each 1.0 cm in diameter, one at the bottom of a side wall of the chamber and the other at the top of the opposite side wall. A gas mixture supply system and gas evacuation system were connected to the lower and upper holes, respectively. The temperature in the chamber was maintained at 23 to 24 °C. The rats were allowed to acclimatize to the chamber for 120 min, with the chamber semi-open and the rats' breathing room air, basal DA being measured and confirmed to be stable at this time. The BP and DA level monitoring tubes were bundled together in a group by a rotating joint (Swivel unit™, SSU-20, Eicom, Kyoto, Japan) and passed through the top panel of the chamber. The gas chamber was then sealed and a gas mixture containing 20% oxygen and 80% nitrogen was supplied to the airtight chamber at the rate of 10 l/min for baseline sampling. After obtaining baseline values of systolic BP (SBP), HR and DA over 60 min under freely moving, awake conditions, the rats were exposed to progressive hypoxia, the oxygen concentration in the chamber being measured continuously using a multi-gas monitor (Narcotica™, HC-510, FUKUDA DENSHI CO., LTD., Tokyo, Japan). The oxygen concentration in the chamber was decreased to 10% within 5 min (descent stage), maintained at 10% for 10 min (fixed stage), and then returned to 20% over 5 min (recovery stage). SBP and HR were sampled at a total of 6 time points during this period: at the attainment of F_I_O_2_ = 0.15 and immediately after the attainment of F_I_O_2_ = 0.1, (descent stage), 5 and 10 min after the attainment of F_I_O_2_ = 0.1 (fixed stage), and at the re-attainment of F_I_O_2_ = 0.15 and immediately after the attainment of F_I_O_2_ = 0.2 (recovery stage), for comparison with the baseline. DA values were measured every 5 min by sampling 8 perfusates (1 during the descent stage, 2 during the fixed stage and 5 during the recovery stage), and the values were compared with basal levels. The respiratory frequency of each rat was counted by visual observation for 10 s during each stage, and the corresponding 1 min rate calculated and recorded. For analysis of arterial blood gases, the rats were subjected to a second cycle of similar levels of hypoxic stress an hour after each experiment and 0.5 ml of blood was drawn during the control period and the late fixed stage in the second hypoxic round, to rule out the influence of data sampling on cardiovascular variability. Blood samples were analyzed for pH, PaO_2_ and PaCO_2_ with a gas analyzer (ABL-30™, Radiometer, Denmark). After the study, all rats were euthanized under deep anesthesia with sevoflurane inhalation in a small plastic Tupperware™ container.

### 2.4 Data recording and analysis

The peak value of BP for each cardiac cycle was sampled as the SBP from artifact-free digitized signals at 1000 Hz, using the wavelet method in real-time with an on-line processing system (Sugimura et al., 2006; Nagai et al., 2003). HR was calculated from the number of peaks in the pressure waveform. Data for SBP and HR were analyzed by power spectral analysis using commercially available software for rats (Fluclet™, Dainippon Sumitomo Pharmaceutical Co., Ltd., Osaka, Japan, wavelet method), whereby SBP was automatically separated into two frequency bands: a low frequency band (LF: 0.25–0.75 Hz) and a high frequency band (HF: 0.75–3.0 Hz). The LF band of SBP variability (SBP-LF) is believed to be a good marker of sympathetic activity in peripheral arteries, although some studies have also shown that SBP-LF reflects overall BP regulatory activity (Japundzic et al., 1990; Baujard et al., 1996; Kawaguchi et al., 2005). On the other hand, it is well known that the HF band of HR variability (HR-HF) reflects activity of the cardiac parasympathetic system (Akselrod et al., 1981; Kochs et al., 2001; Suzuki et al., 2003: Pichot et al., 1999; Ivanov et al., 1996; Nagai et al., 2003; Brown et al., 1994; Cerutti et al., 1991; Pagani et al., 1986; Hayano et al., 1994; Hayano et al., 1991; Pomeranz et al., 1985). A short review of wavelet-based signal analysis is presented in Appendix A.

### 2.5 Statistical analysis

All data, expressed as mean +/− SE, were analyzed by SPSS. Student's *t*-test was used to compare mean hemodynamic and autonomic activity between SHR and WKY, while the effects of hypoxia on SBP and HR variables were evaluated by two-way repeated measures ANOVA. The Dunnett method was used for multiple comparisons. *p* < 0.05 was considered significant in all statistical analyses.

## 3 Results

### 3.1 Influence of progressive hypoxia on HR and SBP

**Fig. 1:**
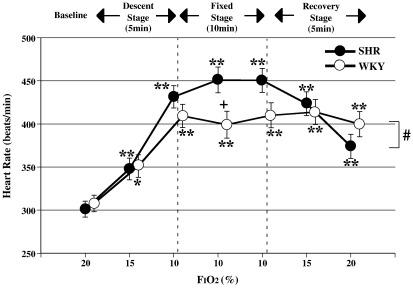
Changes in heart rate with variation in F_I_O_2_, mean +/− SE, *n* = 12 in each group, *: *p* < 0.05 vs baseline (20%), **: *p* < 0.01 vs baseline (20%), +: *p* < 0.05 vs WKY, #: *p* < 0.05 significant interaction (different response between SHR and WKY).

**Fig. 2:**
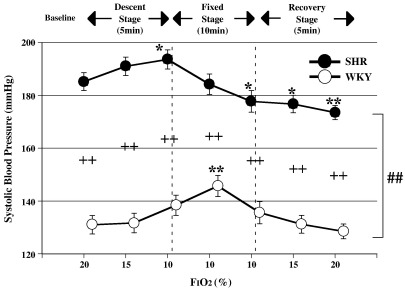
Changes in systolic blood pressure with variation in F_I_O_2_, +/− SE, *n* = 12 in each group, *:*p* < 0.05 vs baseline (20%), **: *p* < 0.01 vs baseline (20%), ++: *p* < 0.01 vs WKY, ##: *p* < 0.01 significant interaction (different response between SHR and WKY).

There were significant differences in the variation patterns of HR and SBP between SHR and WKY (ANOVA for repeated measures: significant interaction of *p* < 0.05 for HR and *p* < 0.01 for SBP, Figs. 1,2). Additionally, baseline SBP in SHR was consistently higher than that in WKY (unpaired *t*-test: *p* < 0.01 vs WKY in SBP, Fig. 2).

Fig. 1 shows the changes in HR of SHR and WKY with hypoxia. The HR of both groups increased significantly during the descent, fixed and recovery stages, compared to baseline values (ANOVA for repeated measures: *p* < 0.05 or *p* < 0.01 in SHR and WKY vs baseline). SHR showed a more remarkable increase in HR than WKY 5 min into the fixed stage (unpaired *t*-test: *p* < 0.05 vs WKY, Fig. 1). SBP of both groups rose transiently from the baseline during the descent and early fixed stages (ANOVA for repeated measures: *p* < 0.05 in SHR and *p* < 0.01 in WKY vs each baseline, Fig. 2), before decreasing back towards baseline values during the late fixed stage. SBP of SHR decreased significantly below baseline in the late fixed and recovery stages (ANOVA for repeated measures: *p* < 0.05 or *p* < 0.01 in SHR vs baseline).

### 3.2 Power spectral analysis of HR and SBP variability by wavelet method

**Fig. 3:**
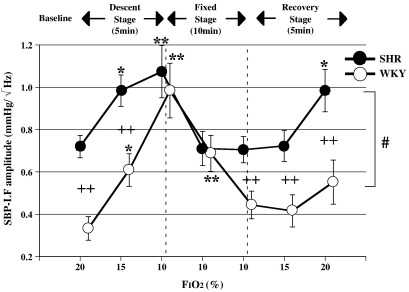
Changes in SBP-LF with variation in F_I_O_2,_ mean +/− SE, *n* = 12 in each group, *: *p* < 0.05 vs baseline (20%), **: *p* < 0.01 vs baseline (20%), ++: *p* < 0.01 vs WKY, #: *p* < 0.05 significant interaction (different response between SHR and WKY).

**Fig. 4:**
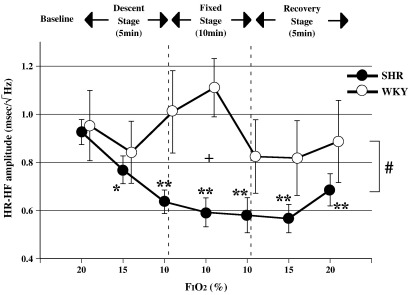
Changes in HR-HF with variation in F_I_O_2,_ mean +/− SE, *n* = 12 in each group, *: *p* < 0.05 vs baseline (20%), **: *p* < 0.01 vs baseline (20%), +: *p* < 0.05 vs WKY, #: *p* < 0.05 significant interaction (different response between SHR and WKY).

There were significant differences in the variation patterns of SBP-LF and HR-HF between SHR and WKY (ANOVA for repeated measures: significant interaction of *p* < 0.05 for SBP-LF, HR-HF, Figs. 3, 4). In addition, baseline values of SBP-LF in SHR were consistently higher than those in each WKY (Unpaired *t*-test: *p* < 0.01 vs WKY in SBP-LF, Fig. 3).

Fig. 3 shows the SBP-LF amplitude component in SHR and WKY. There was a significant difference in baseline SBP-LF amplitude between the two groups. The SBP-LF amplitude of both groups increased significantly during the late descent and early fixed stages (ANOVA for repeated measures: *p* < 0.05 or *p* < 0.01 in SHR and WKY vs baseline). In addition, the SBP-LF amplitude in WKY increased more markedly than in SHR during the late descent stage, this difference between the 2 groups disappearing with the attainment of FiO_2_ = 1. (Unpaired *t*-test: *p* < 0.01 vs WKY in SBP-LF, Fig. 3). The HR-HF amplitude of SHR showed a significant decrease during the descent, fixed and recovery stages (ANOVA for repeated measures: *p* < 0.05 or *p* < 0.01 in SHR vs baseline, Fig. 4), while the HR-HF amplitude of WKY rats showed an upward trend during the early fixed stage, and a downward trend during the late fixed stage.

### 3.3 Striatal DA measurement by in vivo microdialysis and HPLC-ECD

**Fig. 5:**
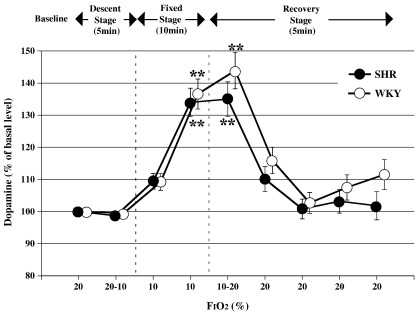
Changes in striatal dopamine levels with variation in F_I_O_2,_ mean +/− SE, *n* = 12 in each group, **: *p* < 0.01 vs baseline (20%).

There was no significant difference in the DA patterns between the two groups. In both groups, the percentage of DA increased significantly during the late fixed and early recovery stages (ANOVA for repeated measures: *p* < 0.01 in SHR and WKY vs baseline, Fig. 5).

### 3.4 Respiratory frequency and blood gas analysis

Respiratory frequency increased significantly in SHR and WKY with development of hypocapnic hypoxia with respiratory alkalosis during the late fixed stage (Table 1).

## 4 Discussion

The most interesting finding of this study is that the cardiac parasympathetic nervous activity of SHR, but not WKY, was inhibited in a sustained manner, as indicated by the decrease in HR-HF just after the beginning of acute progressive hypoxia. It is conceivable that the decrease in parasympathetic activity in SHR contributes markedly to the persistent increase in HR during all stages and to the transient elevation in SBP during the descent and early fixed stages, along with transient accentuation of sympathetic activity. While it is well known that vagal activity is naturally diminished in hypertensive humans and animals (Guzzetti et al., 1988; Friberg et al., 1988, 1989), and that baroreceptor control of HR is reduced mainly due to the inhibited vagal component rather than by changes in sympathetic activity (Head, 1994), how additional stress influences the ANS in hypertensive animals has not been entirely clarified. Guzzetti et al. (1988) found that a painful stimulus in essential hypertensive patients induced a parasympathetic inhibitory effect along with a sympathomimetic one. Furthermore, Moriguchi et al. (1992) reported that the performance of mental arithmetic by essential hypertensive patients decreased HR-HF and increased the HR-LF/HF ratio, indicating sympathetic nervous activation. Consequently, it is believed that acute stress in hypertensives, regardless of the kind of stress, inhibits parasympathetic activity, which can become a significant modifier of the hemodynamic response. Hence, although acute stress has an initial sympathomimetic effect followed by a parasympathomimetic effect due to baroreceptor reflexes (BR) (Levy and Zieske, 1969), it is suggested that acute hypoxic stress in hypertensive rats instead triggers a parasympathetic inhibition as demonstrated in our study. One possible mechanism for this finding might be that advanced arteriosclerosis, which is common in hypertensives, makes it difficult for baroreceptors to stretch with BP elevation (Boddaert et al., 2004; Shimada et al., 1985), leading to attenuation/inhibition of the parasympathetic response. Moreover, it is thought that hypoxia itself has a significant inhibitory effect on BR. Ziegler et al. (1995) reported that hypoxia impaired BR, as indicated by the finding that the BR sensitivity (BRS) in humans declined with inhalation of a gas mixture containing 10% oxygen. The findings of our study suggest that the BRS of rats, in particular of SHR, tends to be suppressed by the same mechanism, as shown by the sustained decrease in HR-HF even during BP elevation. Recently, it has been noted that lowering of parasympathetic activity and BRS is indicative of a poor prognosis in patients with serious heart failure (Myredal et al., 2005; Mortara et al., 1997). Autonomic dysfunction, as indicated by the inability to activate effective vagal reflexes, might be partly linked to the sustained decline in HR-HF of SHR with progressive hypoxia, in contrast to WKY, as seen in our study.

On the other hand, SBP of SHR showed a significant decrease during the late fixed and recovery stages in spite of the decrease in HR-HF, contrary to WKY, in whom SBP did not decrease. There are two possible reasons for this phenomenon in SHR under continued hypoxic stress. One is the direct inhibitory effect of hypoxia on the cardiovascular system, and the other is direct inhibition of the CNS with a focus on rostral ventrolateral medulla (RVLM) reticulospinal sympathoexcitatory vasomotor neurons, particularly in SHR (Sun and Reis, 1994; Koganezawa and Terui, 2007). Regarding the former, there are some reports that a fall in BP is induced by systemic hypoxia, which provokes direct vasodilation and cardiodepression (Marshall and Metcalfe, 1988; Hirakawa et al., 1997). Marshall and Metcalfe (1988) proved that the change resulted from direct inhibition of the cardiovascular system by hypoxia, but not via the ANS, since autonomic blocking agents did not prevent this reaction. The biochemical mechanisms for these changes due to hypoxia are probably adenosine-induced vasodilation, adenosine being a by-product of ATP degradation, and the effect of nitric oxide released from vascular endothelial cells, in addition to inhibition of myocardial contractility by ATP depletion (Mian and Marshall, 1995; Jernigan et al., 2004). Myocardial depression and vasodilation of skeletal muscles have also been attributed to elevated serum potassium levels through activation of ATP-sensitive K^+^ channels (Marshall et al., 1993). Irrespective of the mechanism, it is thought that weakened contractile responses of the myocardium and vessels resulting from hypoxia cause the transition from transient elevation to continued lowering of SBP in SHR in the early fixed stage. In addition, it is suggested that the inherent arteriosclerotic inelasticity of vessels in SHR also prolongs the decrease in SBP, even after relief from hypoxic stress, because arteriosclerosis has been shown to bring about weakening of sympathetically mediated vasoconstriction (Boddaert et al., 2004).

Hypoxia also directly inhibits the cerebral vasomotor center as well as the peripheral cardiovascular system in SHR (Sun and Reis, 1994; Koganezawa and Terui, 2007). Sun and Reis (1994) reported that RVLM reticulospinal sympathoexcitatory vasomotor neurons might be selectively and directly stimulated by hypoxia in chemodeafferented rats, in addition to mediation of sympathoexcitation associated with arterial chemoreceptor stimulation. In the latest finding, Koganezawa and Terui (2007), using anesthetized and vagotomized rabbits, found that there were three groups of barosensitive reticulospinal neurons in the RVLM, differentiated according to their responses to hypoxic stimulation. The authors also reported that one group of the neurons showed inhibitory responses to hypoxic gas (3% O_2_–97% N_2_) inhalation for 1 min while other groups did not. Taken together, hypoxic stress could directly depress the RVLM reticulospinal sympathoexcitatory vasomotor neurons, although the output might show a biphasic alteration under experimental conditions. Recently, it has also been proposed that premotor neurons for the cardiac sympathetic nerve are located in the raphe nucleus, these being suggested to convey information from the hypothalamus to the spinal cord independent of the baroreceptor reflex (Cao and Marisson, 2003; Cao et al., 2004). On the other hand, it has also been reported that most neurons in the brain exposed to hypoxic stress reduce their metabolic requirements by decreasing their activity and thereby adapt to the harsh environment (Neubauer and Sunderram, 2004). Consequently, as shown by the disappearance of the increase in SBP-LF during the late fixed stage, it is thought that inhibition of sympathetic activity in efferent neurons is another factor contributing to the decrease in SBP. As far as we know, no studies have attempted to clarify the differences in the circulatory center responses to acute progressive hypoxic stress between SHR and WKY. Our results show that the differences in the responses of the two types of rats are influenced by CNS-mediated effects such as inherent sympathetic hyperactivity in SHR themselves (Fig. 3), the greater magnitude of the sympathetic response in WKY during the early hypoxic stage (Fig. 3), and contrasting parasympathetic responses between the two types of rats during all stages (Fig. 4).

We are unable to disregard the influence of hypocapnia in our study. Hirakawa and Murasato (1997) and Murasato et al. (1998) reported that parasympathetic nerve activity is changed in parallel with PaCO_2_ under hypoxic stress (F_I_O_2_ = 0.1), after 30 min of the hypoxic insult. In addition, they also showed that the phenomenon is blocked by pre-treatment with atropine sulfate, showing that the HR-HF component reflects parasympathetic nervous system activity. On the basis of the above findings, we carried out our study in SHR under acute progressive hypoxia for 20 min, and observed that cardiac parasympathetic activity in SHR was significantly inhibited by hypoxia during all stages, as shown by the decrease in HR-HF. We believe that hypocapnia is a modifier of this finding, particularly in SHR. It is thought that involvement of hypocapnia in ANS activity progresses with time, resulting in the decrease of HR-HF in SHR and its downward tendency in WKY. This mechanism is thought to be the dominant cause of continuance of tachycardia during the late hypoxic phase.

We measured striatal DA as an index of hypoxic stress in the CNS because the increase of extracellular DA, especially in the striatum and the hippocampus, that occurs in oxygen-deprived conditions is known to have harmful influences, including abetment of irreversible neural death (Nakajima et al., 1996; Busto et al., 1985; Globus et al., 1988; Globus et al., 1987; Masuda and Ito, 1993; Hedner and Lundborg, 1979). Increase of DA could lead to motor disturbances, behavioral abnormalities and learning disabilities over the long run (Gross et al., 1986; Erinoff et al., 1979; Hagberg et al., 1981). Striatal DA levels in this study showed a transient increase in both types of rats from the late fixed to the early recovery stages, returning to baseline after withdrawal of the hypoxic stress. Hence, in our study protocol, hypoxia was unlikely to have increased the DA level by destruction of DA neurons, since the DA dynamics showed reversible changes. Instead, it is thought that the increased DA levels were mainly caused by persistence of DA due to decreased reuptake resulting from weakening of DA transporter activity (Nakajima et al., 1996; Orset et al., 2005) and decreased degradation due to lowered monoamine oxidase activity (Nakajima et al., 1996). In addition, Sutoo et al. (1989, 1993) and Sutoo and Akiyama (2002) reported that changes in serum calcium kinetics due to stress, such as cold stimulation, electric shock and psychophobia, have an influence on the regulation of DA levels in the striatal body and nucleus accumbens. Based on this finding, we conducted our present study to assess the regulation of striatal DA levels through changes in serum calcium kinetics resulting from acute hypoxic stress. However, it is too early to draw any conclusions regarding the linkage between striatal DA levels and ANS activity because the serum calcium level was not measured. Further studies are required to fully elucidate the linkage.

In the present study, we performed power spectral analyses of HR and SBP variabilities using the WT method (Fluclet™) because of its high time resolution, in addition to its being easier to perform and less invasive. (Akselrod et al., 1981; Kochs et al., 2001; Suzuki et al., 2003; Pichot et al., 1999; Ivanov et al., 1996). This method has been considered to provide markedly better quantitative analysis of cardiovascular variability than the fast Fourier transform (FFT) during autonomic nervous adaptations induced by external agents or some sort of stress with two main advantages. First, WT allows a temporally localized sliding analysis of signals using a known function that is called the mother wavelet. When the balance of the autonomic nervous equilibrium is instantaneously modified in clinical situations, such as acute progressive hypoxia, the WT method can be used to assess the status of HR and BP variabilities at any time point. Second, the shape of the WT analysis equation differs from the fixed sinusoidal shape of FFT, and can be customized to fit the shape of the analyzed signal, allowing a better quantitative measurement (Appendix A). These features allow for high time resolution and applicability to non-stationary data, which are needed for accurate autonomic assessment under conditions of acute progressive hypoxia.

In conclusion, our study demonstrates the time course of changes in the ANS and cardiovascular functions in conscious SHR and WKY during acute progressive hypoxia. Our results suggest that acute hypoxic stress in SHR inhibits cardiac parasympathetic activity, probably through reduction of baroreceptor sensitivity itself. This phenomenon might result in a marked change in circulation, particularly in HR and the suppression of circulatory autoregulatory mechanisms. On the other hand, striatal DA levels that could have harmful influences on the CNS in oxygen-deprived conditions showed a transient increase in SHR and WKY, the levels of increases being similar in both groups.

## Figures and Tables

**Table 1:** Changes in respiratory frequency and arterial blood gas analysis

			Baseline	During the late fixed stage
Respiratory frequency (breaths/min)		SHR	87.3 ± 2.2	192.6 ± 9.7 **
		WKY	76.2 ± 2.3	143.2 ± 10.2 *
Arterial blood gas analysis	pH	SHR	7.389 ± 0.022	7.582 ± 0.021 *
		WKY	7.403 ± 0.017	7.550 ± 0.016 *
	PaCO_2_ (mm Hg)	SHR	37.4 ± 0.60	16.7 ± 1.8 **
		WKY	38.4 ± 0.10	21.7 ± 1.3 **
	PaO_2_ (mm Hg)	SHR	94.7 ± 1.9	37.8 ± 4.5 **
		WKY	95.2 ± 1.4	42.8 ± 4.0 **

Data are expressed as the mean ± SE. * *p* < 0.01 vs baseline or ** *p* < 0.001 vs baseline. Respiratory frequency increased significantly in SHR and WKY with development of hypocapnic hypoxia, followed by respiratory alkalosis during the late fixed stage.

## Appendix A

### Analysis of heart rate and blood pressure variability

We utilized the Fluclet software (Dainippon Sumitomo Pharma Co., Ltd, Osaka, Japan.) for the continuous WT to study the dynamic variation of autonomic nervous activity in rats, exploring heart rate and blood pressure variability over very short time intervals. WT searches the nature of signals by adapting the function *ψ* (*t*), which is called the mother (basic or analyzing) wavelet, and which localizes in both time and frequency domain. A mother wavelet slides along the time direction, and contracts or stretches in the high or low frequency region, respectively. So, continuous WT is defined by the following equation:

(1) $$W_{f}(\alpha ,b)=\int_{-\infty }^{\infty }\frac{1}{\sqrt{\left|\alpha \right|}}\overset{―}{\psi \left(\frac{t-b}{\alpha \alpha }\right)}f(t)dt$$

where *b* is a time shift parameter, *α* is a scale parameter of elasticity, with 1 / *α* corresponding to frequency, *f*(*t*) is an input signal, the symbol ^−^ indicates complex conjugates, and *W* is called the wavelet coefficient. Moreover, with WT it is possible to obtain 3-dimensional- or contour-plots by plotting *W* for the pair of parameters (*b*, 1 / *α*). This plot is useful for observing changes in dominant frequency ranges with time. Gabor's wavelet was used as the mother wavelet in this fluctuation analysis (Akay, 1995). This wavelet is believed to be suitable for frequency analysis according to the equation:

(2) $$\psi (t)=\frac{1}{2\sqrt{\pi \sigma }}e^{-\frac{t^{2}}{\sigma^{2}}}e^{-1t}$$

where *σ* is a positive constant. The absolute value of *W*_*f*_ (*α*, *b*) is regarded as the potency at time *b* and scale *α*, as in FFT. The relationship between the scale *α* and the real (local) frequency *f* is *f*  =  *f*_0_ / *α*, where *f*_0_ is the central frequency of the mother wavelet. For Gabor's wavelet, *f*_0_ = 1 / (2*π*) at *σ* = 8 was obtained by applying Fourier transformation to formula (II). Formula (I) was digitalized for computer processing. This digitalization was noted as being different from the discrete WT. In digitalized continuous WT, 100 mother wavelets were constructed to obtain one periodogram. The RR intervals extracted from a continuous electrocardiogram record were interpolated every 100 ms by a cubic spline. The digitalized Eq. (1) was evaluated at different values of time shift *b*, successively shifting over 100 ms, or one interpolated pseudo-sample for each subsequent evaluation, and the periodogram was obtained using the absolute value of wavelet coefficients. The resolution of overlapped frequency components was applied to the periodograms to estimate the number and peak of overlapped components in the periodogram. LF (low frequency) and HF (high frequency) components were defined as the resolved components whose peaks were within the following ranges: 0.27–0.74 Hz and 0.74–2.00 Hz, respectively. In the present study, the spectral components HR-HF were produced for every 5-second interval with the continuous WT, and the SBP-LF was also calculated. SBP-LF gives mainly an estimate of sympathetic activity with some influence from the vagal nervous system; HR-HF reflects solely vagal activity (Akselrod et al., 1981; Pagani et al., 1986; Pomeranz et al., 1985; Tan et al., 2003).
